# Impact of Internet Use on Cognitive Decline in Middle-Aged and Older Adults in China: Longitudinal Observational Study

**DOI:** 10.2196/25760

**Published:** 2022-01-24

**Authors:** Xinyue Yu, Aruhan Mu, Xiang Wu, Liqin Zhou

**Affiliations:** 1 School of Medicine and Health Management Huazhong University of Science and Technology Wuhan China

**Keywords:** internet use, cognitive decline, China, fixed-effects analysis

## Abstract

**Background:**

Given that cognitive decline lacks effective treatment options and has severe implications for healthy aging, internet use may achieve nonpharmacological relief of cognitive decline through cognitive stimulation and social engagement.

**Objective:**

This longitudinal study aimed to investigate the relationship between the diversity, frequency, and type of internet use and cognitive decline, and to provide theoretical support and suggestions for mitigating cognitive decline in middle-aged and older adults.

**Methods:**

Data were obtained from a total of 10,532 survey respondents from the China Family Panel Studies database from wave 3 (2014) and wave 5 (2018) of the survey. Cognitive function was measured using vocabulary tests, and internet use was categorized into five aspects: study, work, socializing, entertainment, and commercial-related activities. Associations between the diversity, frequency, and type of internet use and cognitive decline were estimated by controlling for demographic variables and health status risk factors through fixed-effects models.

**Results:**

After controlling for demographic and health status risk factors, the type and frequency of internet use were found to be associated with cognitive functioning during the subsequent 4-year period, and different types of internet use had different effects on cognitive decline. Frequency of internet use of at least once a week for study (β=0.620, 95% CI 0.061 to 1.180; *P*=.04), work (β=0.896, 95% CI 0.271 to 1.520; *P*=.01), and entertainment (β=0.385, 95% CI –0.008 to 0.778; *P*=.06), as well as less than once a week for social purposes (β=0.860, 95% CI 0.074 to 1.650; *P*=.06), were associated with better cognitive function. Frequency of internet use of less than once a week for commercial-related activities (β=–0.906, 95% CI –1.480 to –0.337; *P*=.005) was associated with poorer cognitive function. Using the internet for more than one type of activity (β=0.458, 95% CI 0.065 to 0.850; *P*=.03) and at least once a week (β=0.436, 95% CI 0.066 to 0.806; *P*=.02) was associated with better cognitive function.

**Conclusions:**

This study shows that breadth and depth of internet use are positively associated with cognitive function and that different types of internet use have different roles in cognitive decline. The importance of the internet as a nonpharmacological intervention pathway for cognitive decline is emphasized. Future research could explore specific mechanisms of influence.

## Introduction

Cognitive decline is an irreversible process of pathological changes in the brain that often starts in individuals aged 45 to 60 years [1]. The aggravation of cognitive decline is likely to lead to dementia as well as physical disability and death [2]. Some studies have shown that almost 20% of Chinese people over 60 years of age have mild cognitive decline and develop dementia at a rate of 6% every year [3]. Although there is no effective treatment for cognitive decline, neuroscience and cognitive aging studies have shown that patients with cognitive decline retain some cognitive abilities and plasticity [4,5]. The results of an intervention evaluation on the impact of global dementia interventions showed that interventions that delay disease onset and progression by 1 year would reduce the incidence of dementia by 9.2 million cases in 2025 [6]. Cognitive decline is not only a significant burden on society and the economy but also carries the pressure of informal care costs and health resources. Therefore, the use of nonpharmacological interventions for middle-aged and older adults aged 45 years and above is of great significance in reducing the occurrence of cognitive decline, slowing down cognitive decline, and even reversing the disease [7].

In studies exploring risk factors for cognitive decline, demographic variables, individual health status, and social factors are largely taken into account, and the conclusions reached are largely consistent. Among them, demographic variables, such as age and education, are risk factors for cognitive decline. Physical diseases, such as cardiovascular disease and stroke [8]; unhealthy behaviors, such as smoking and alcohol consumption [9-12]; and social isolation, loneliness, depression, and other conditions [13-16] can also harm cognitive function in middle-aged and older adults. The World Health Organization (WHO), in its Guidelines for Mitigating the Risk of Cognitive Decline and Dementia, recommends reducing the risk of cognitive decline by living a healthy lifestyle. However, there are individual differences among older people, including reduced mobility, physical deterioration, social isolation, and differences in their surroundings, resulting in limited access and low availability of resources [17]. The increase in internet penetration has allowed internet use to gradually penetrate middle and older age groups [18]. The researchers are interested in the impact of internet use as a cognitive stimulus on middle-aged and older adults, providing an opportunity to mitigate cognitive decline. This measure is highly actionable.

Over the past few years, researchers have conducted several studies on the effects of internet use on cognition. Research shows that using the internet can improve the choice ability of older adults with cognitive decline. Training older adults to use computers and the internet can positively impact their quality of life [18]. Owning electronic devices can reduce cognitive decline in older adults [17,19]; older adults’ use of email, serious video games, and virtual reality may mitigate cognitive decline [19,20]. Learning and using online social networking sites can be used as interventions to maintain or enhance cognitive function in older adults [21]. The use of social networking sites by older adults will improve perceived social support and connection, and reduce perceived social isolation [22]. Previous studies mainly measured internet use as the use of email or digital devices. They used cross-sectional data more often to determine the impact of internet use on cognitive decline. In the Chinese context, email is hardly representative of internet use. China Family Panel Studies (CFPS) is a biennial prospective observational study; this study used 2014 data as the baseline and 2018 data as the follow-up to investigate the following: (1) whether five types of internet use were independently associated with cognitive decline in middle-aged and older adults and (2) whether frequency and diversity of internet use were independently associated with cognitive decline in middle-aged and older adults.

## Methods

### Sample and Data Collection

**Figure 1:**
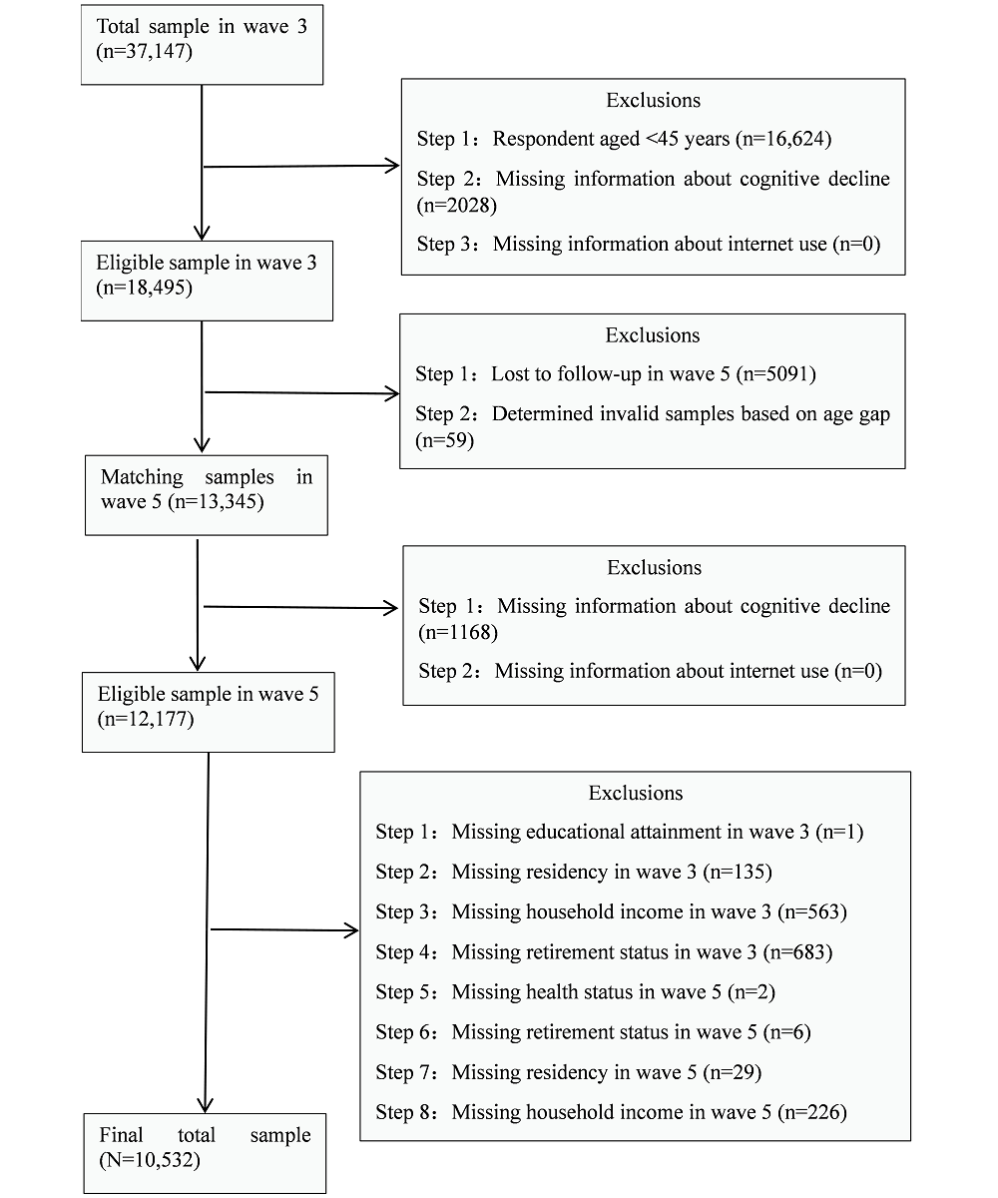
Flowchart showing respondent screening.

The data for this study were from the CFPS database survey data that were collected in 2014 (wave 3) and 2018 (wave 5) [23]. The 2 years of survey data were collected using the same cognitive test questions, and both tested the internet use module. The CFPS baseline (2010) sample covers 25 provinces, municipalities, or autonomous regions, representing 95% of China’s population. The researchers who conducted the 2010 baseline survey interviewed a total of 14,960 households and 42,590 individuals, and they launched a long-term follow-up survey of individual samples. Data cleaning resulted in a sample of 37,147 respondents. This study consisted of 10,532 adults aged 45 years or older in 2014. We used data from wave 3 in 2014 as baseline data, and we used data from wave 5 in 2018 as follow-up data, which had been collected after a 4-year follow-up period. Cognitive function was tested using the same questions at baseline testing and during follow-up. We further excluded samples according to the following criteria: (1) respondents aged less than 45 years at baseline (n=16,624), (2) no information provided on cognitive function (n=3196) or internet use (n=0), (3) loss of respondents after matching the two waves (n=5091), and (4) missing sample of control variables (n=1704). A total of 10,532 respondents were finally screened. Figure 1 illustrates the exclusion criteria and the process of respondent screening.

### Measures

#### Dependent Variable

Cognitive function was the dependent variable. In order to assess the level of cognitive function, we used a vocabulary test. The test, which consisted of 34 Chinese characters, sought to measure one’s vocabulary by testing a respondent’s ability to recognize difficult characters; the score ranged from 0 to 34 [24].

#### Independent Variables: Internet Use

In this study, we examined internet use from the following three aspects:

1. Type of internet use. This item included the frequency of using the internet for the following five topics: study, work, socializing, entertainment, and commercial-related activities. For example, “In general, how frequently do you use the internet to study, such as searching for study materials or online study courses?” The survey participants selected a frequency to reflect their use of the internet for each purpose. The variable was coded as 0 (none), 1 (less than once a week), or 2 (at least once a week).
2. Diversity of internet use types. This represented the participant’s total number of different internet use types, which were coded as 0 (none), 1 (one type), or 2 (more than one type).
3. Frequency of internet use. This represented the participant’s maximum frequency of internet use. We merged the groups who reported a frequency of more than once a week and recoded the responses as “at least once a week.” Groups who reported a frequency of less than once a week were combined, and the responses were recoded as “less than once a week.” Therefore, the variable was coded as 0 (none), 1 (less than once a week), or 2 (at least once a week).

#### Control Variables

A set of variables previously revealed to be associated with internet use and cognitive function (ie, gender, age, educational attainment, marital status, household income, residency, health status, and retirement status) were controlled to provide more compelling evidence by reducing the amount of confounding influence they might have on the effects of internet use. Gender, marital status, residency, and retirement status were defined as dichotomous variables (gender: 0 [man] or 1 [woman]; marital status: 0 [single] or 1 [married or living as married]; residency: 0 [rural] or 1 [urban]; retirement status: 0 [no] or 1 [yes]). Age was measured in years, and educational attainment was measured by asking the respondent to indicate the highest level of education completed: 1 (illiterate), 2 (primary school), 3 (junior high school), 4 (senior high school), or 5 (college and above). Health status was measured according to respondents’ self-rated physical health status: 1 (poor), 2 (fair), 3 (good), 4 (very good), or 5 (excellent). Household income was the total income from all types of work and nonwork for all household members divided by the number of household members; this was log-transformed because of its highly skewed distribution.

### Data Analysis

Descriptive analyses were conducted to summarize the demographic characteristics of the study sample. Participants’ characteristics were summarized using mean (SD) for continuous variables and proportions for categorical variables. We aimed to determine whether there was a significant difference in the highest frequency and diversity of internet use between different groups. In this case, the chi-square test was used for categorical variables, and we used the Kruskal-Wallis test for all continuous variables.

We used the Hausmann test, which is more suitable for panel data processing, to implement the fixed-effects model, taking into account both individual and time effects of the sample. The measurement model is constructed as follows:

*CognitionFunction_it_* = *β_1_Internet_it_* + *β_2_x_it_* + *α_i_* + *μ_t_* + *ε_it_*

*CognitionFunction_it_* represents the score of individual *i* on a word test of cognitive function at time *t*; *Internet_it_* represents individual *i*’s purpose, frequency, and diversity of different internet uses at time *t*; *x_it_* represents characteristics of individual *i* over time, including marital status, retirement status, income, and general health; *α_i_* represents characteristics of individual *i* that do not change over time, including gender, education level, and possible unobservable effects; *μ_t_* represents the effect that time *t* does not vary from individual to individual; and *ε_it_* represents the error term.

The main model consisted of three regression equations measuring the following three relationships: the relationship between the diversity of respondents’ internet use and their cognitive function, the relationship between respondents’ maximum frequency and their cognitive function, and the relationship between respondents’ frequency of internet use for different purposes and their cognitive function. Control variables were included throughout the analyses. All analyses were performed using R (version 4.0.2; The R Foundation).

## Results

### Descriptive Statistics

The basic characteristics of the whole sample population (N=10,532) at baseline for this study (ie, wave 3) are shown in Table 1. The mean age of the respondents was 58.7 (SD 9.3) years, 5405 (51.3%) participants were female, and 3526 (33.5%) participants were retired. A total of 10,202 (96.9%) participants had no college education, 9518 (90.4%) were married or cohabiting, and 6456 (61.3%) considered their health to be good. The mean household income of the participants was ¥12,737.30 (SD ¥16,569.10; mean US $1999.39, SD US $2600.87). Moreover, we found that all covariates were significantly associated with the frequency of internet use. In addition, we observed that all covariates were significantly associated with the frequency of respondents’ internet use by chi-square test and Kruskal-Wallis test. This is consistent with the results of previous studies.

**Table 1:** Sample characteristics of respondents at baseline.

Variable		Value (N=10,532)	Frequency of internet use		Diversity of internet use	
			Chi-square^a^ (*df*) or Kruskal-Wallis^b^ (*df*)	*P* value	Chi-square^a^ (*df*) or Kruskal-Wallis^b^ (*df*)	*P* value
**Gender, n (%)**						
	Male	5127 (48.7)	39.7^c^ (2)	<.001^c^	38.9 (2)	<.001
	Female	5405 (51.3)				
Age (years), mean (SD)		58.7 (9.3)	393.0 (2)	<.001	406.0 (2)	<.001
**Educational attainment, n (%)**						
	Illiterate	4001 (38.0)	2077.7 (8)	<.001	2227.0 (8)	<.001
	Primary school	2459 (23.4)				
	Junior high school	2576 (24.5)				
	Senior high school	1166 (11.1)				
	College and above	330 (3.1)				
**Marital status, n (%)**						
	Single	1014 (9.6)	10.4 (2)	.006	10.7 (2)	.005
	Married or living as married	9518 (90.4)				
**Residency, n (%)**						
	Rural	5916 (56.2)	508.7 (2)	<.001	508.4 (2)	<.001
	Urban	4616 (43.8)				
**Retirement status, n (%)**						
	No	7006 (66.5)	33.6 (2)	<.001	43.8 (2)	<.001
	Yes	3526 (33.5)				
Household income (¥^d^), mean (SD)		12,737.30 (16,569.10)	547.0 (2)	<.001	547.0 (2)	<.001
**Health status, n (%)**						
	Poor	2297 (21.8)	126.1 (8)	<.001	128.3 (8)	<.001
	Fair	1179 (16.9)				
	Good	3760 (35.7)				
	Very good	1630 (15.5)				
	Excellent	1066 (10.1)				

^a^Chi-square tests were performed for gender, educational attainment, marital status, residency, retirement status, and health status.

^b^Kruskal-Wallis tests were performed for age and household income.

^c^The statistical test value and the *P* value for a group of variables is listed in the top row of that group.

^d^A currency exchange rate of US $1=¥6.37 is applicable.

### Internet Use Among Older Adults

Table 2 shows data from two waves regarding the diversity, frequency, and type of internet use among the middle-aged and older adult population (N=10,532). The participation rates show that respondents did not use the internet to a very high degree, while in 2018, the usage rates saw a slight increase of 14.9%. However, there were still more than 75% of middle-aged and older adults who did not use the internet. In 2014, the lowest rate of internet use was for commercial-related activities (n=245, 2.3%), and the highest rate of internet use was for entertainment (n=694, 6.6%). In 2018, the lowest rate of internet use was for work (n=620, 5.9%), and the highest rate of internet use was for socializing (n=2131, 20.2%). The 2-year comparison shows that the middle-aged and older adult respondents who used the internet for socializing had the highest growth rate of 15.3%, while those who used the internet for work had the lowest growth rate of 2.3%.

**Table 2:** Internet use type, frequency, and diversity by respondents for each of the two waves.

Internet use					Participants (N=10,532), n (%)				
					Wave 3 (2014)		Wave 5 (2018)		
**Type**									
	**Study**								
		None		9959 (94.6)		9609 (91.2)			
		Less than once a week		104 (1.0)		220 (2.1)			
		At least once a week		469 (4.4)		703 (6.7)			
	**Work**								
		None		10,149 (96.4)		9912 (94.1)			
		Less than once a week		70 (0.7)		84 (0.8)			
		At least once a week		313 (3.0)		536 (5.1)			
	**Socializing**								
		None		10,012 (95.1)		8401 (79.8)			
		Less than once a week		102 (1.0)		182 (1.7)			
		At least once a week		418 (4.0)		1949 (18.5)			
	**Entertainment**								
		None		9838 (93.4)		8449 (80.2)			
		Less than once a week		160 (1.5)		223 (2.1)			
		At least once a week		534 (5.1)		1860 (17.7)			
	**Commercial-related activities**								
		None		10,287 (98.2)		9631 (91.5)			
		Less than once a week		191 (1.8)		507 (4.8)			
		At least once a week		54 (0.5)		394 (3.7)			
**Diversity**									
	None				9651 (91.6)		8081 (76.7)		
	One type				176 (1.7)		401 (3.8)		
	More than one type				705 (6.7)		2050 (19.5)		
**Frequency**									
	None				9651 (91.6)		8081 (76.7)		
	Less than once a week				73 (0.7)		105 (1.0)		
	At least once a week				808 (7.7)		2346 (22.3)		

In 2018, as compared to 2014, there was a 2.1% increase in the number of subjects participating in one type of internet activity and a 12.8% increase in the number of subjects participating in more than one kind of online activity. The participation of people 45 years of age and older in internet activities increased in breadth. The subjects’ frequent participation (ie, at least once a week) in online activities increased by 14.6% in 2018, as compared to 2014, while participation of less than once a week increased by 0.3%. Internet activity participation by people aged 45 years and over gradually shifted to deeper involvement.

### Relationship Between Internet Use and Cognitive Function

Table 3 outlines the contemporary association between cognitive function and the diversity, frequency, and type of internet use between waves 3 and 5 with other time-varying confounders controlled. We included covariates in a fixed-effects model in which residency and health status emerged as significantly associated with cognitive function. Urban users were associated with better cognitive function compared to rural users (β=0.687, *P*=.03). Respondents in better health had better cognitive functioning compared to those in poor health, with a health status rating of “excellent” being most associated with better cognitive functioning (β=0.602, *P*=.01). Our variables were all categorical variables, and the rating “none” represents the baseline level. The results of the fixed-effects model showed a relationship between cognitive function and frequencies of internet use of “less than once a week” and “at least once a week,” as compared to a frequency of “none.” Participating in multiple types of internet activities and engaging in internet activities at least once a week were associated with better cognitive function in middle-aged and older adults (β=0.458, *P*=.03; β=0.436, *P*=.02), whereas participating in a single type of internet activity and using the internet less than once a week were not associated with cognitive function. Using the internet at least once a week for study and work was associated with better cognitive function (β=0.620, *P*=.04; β=0.896, *P*=.01), whereas using the internet less than once a week for study failed to suggest a relationship and using the internet less than once a week for work still predicted better cognitive function (β=0.955, *P*=.11). Using the internet less than once a week for socializing and using it at least once a week for entertainment were associated with better cognitive function (β=0.860, *P*=.06; β=0.385, *P*=.06), but no relationship could be observed between better cognitive function and using the internet at least once a week for socializing and less than once a week for entertainment. Using the internet less than once a week for commercial-related activities was associated with worse cognitive function (β=–0.906, *P*=.005). No relationship could be observed between using the internet more than once a week for commercial-related activities and better cognitive function.

**Table 3:** Associations between cognitive function and internet use diversity, frequency, and type using fixed-effects regression.

Independent variables: internet use^a,b^					Dependent variable: cognitive function		
					β (95% CI)		*P* value
**Diversity**							
	None				Reference		N/A^c^
	One type				0.319 (–0.328 to 0.966)		.33
	More than one type				0.458 (0.065 to 0.850)		.03
**Frequency**							
	None				Reference		N/A
	Less than once a week				0.267 (–0.893 to 1.430)		.64
	At least once a week				0.436 (0.066 to 0.806)		.02
**Type**							
	**Study**						
		None		Reference		N/A	
		Less than once a week		0.529 (–0.202 to 1.260)		.23	
		At least once a week		0.620 (0.061 to 1.180)		.04	
	**Work**						
		None		Reference		N/A	
		Less than once a week		0.955 (–0.087 to 2.000)		.11	
		At least once a week		0.896 (0.271 to 1.520)		.01	
	**Socializing**						
		None		Reference		N/A	
		Less than once a week		0.860 (0.074 to 1.650)		.06	
		At least once a week		0.004 (–0.364 to 0.372)		.98	
	**Entertainment**						
		None		Reference		N/A	
		Less than once a week		0.512 (–0.242 to 1.270)		.21	
		At least once a week		0.385 (–0.008 to 0.778)		.06	
	**Commercial-related activities**						
		None		Reference		N/A	
		Less than once a week		–0.906 (–1.480 to –0.337)		.005	
		At least once a week		–0.566 (–1.280 to 0.152)		.15	

^a^All models controlled for time-varying variables, including wave, gender, educational attainment, marital status, household income, residency, health status, and retirement status.

^b^There were 21,064 observations, and R^2^=0.002.

^c^N/A: not applicable; this row is the reference value.

## Discussion

### Principal Findings

This study was nationally representative and showed longitudinal protective associations between different internet use dimensions and cognitive decline among the older adult population in China. The global population is aging rapidly, and China is a country with a large older adult population, of which 177 million are over 65 years of age. According to WHO estimates, the proportion of the world’s population over 60 years of age will double between 2000 and 2050, from 11% to 22% [7]. Therefore, as the aging population is growing, the use of nonpharmacological interventions for middle-aged and older adults aged 45 years and above is of great significance in reducing the incidence of cognitive decline, delaying cognitive decline, and reversing the disease in middle-aged and older adults. As cognitive decline incidence continues to rise, the morbidity age becomes lower and the medical burden continues to increase. What is more, the disease is more likely to develop into dementia without effective drug treatment. As of June 2019, the number of internet users in China reached 854 million. The proportion of internet users aged 50 years and above increased from 12.5% to 13.6% at the end of 2018, and internet penetration continued among middle-aged and older adult age groups. Internet use offers opportunities and high accessibility to mitigate cognitive decline among older adults. Based on a nationally representative survey by CFPS, we found that the correlation between internet use and cognitive decline is still present in China. The different effects of the internet use dimensions on cognitive decline are worth discussing.

We found that internet users had better cognitive function compared to nonusers at the 4-year follow-up. Evidence in other settings includes using five periods of data from the English Longitudinal Study of Aging (ELSA) database, with cognitive function as the dependent variable and whether they used the internet or email as the independent variable, to conclude that internet use helped reduce the number of people aged 50 to 89 years with cognitive decline [20]. Although there is no definitive mechanism that affects cognitive decline, there are two widely accepted mechanisms in the research to explain this. The first is the cognitive reserve hypothesis [25-27], which views internet use as a cognitively stimulating activity that uses the brain’s neural networks to decrease brain damage. The second is the stress hypothesis [28], which views the internet as a form of social engagement, where the sense of belonging and constructed social networks generated by the activity stimulate brain evolution and functional development. Failure to adapt to stress, which is associated with the pathogenesis of dementia, increases an individual’s glucocorticoid levels; this increase in glucocorticoids leads to hippocampal damage, which can impair an individual’s cognitive function [29]. These hypotheses explain, to some extent, the results of this study.

We built upon previous research to verify that different residency and health statuses in middle-aged and older adults are associated with different cognitive functions. Older adults who live in urban settings and are in good health are less likely to experience cognitive decline. Diversity and frequency of internet use have differential relationships on cognitive decline. More extensive and frequent use of the internet as a cognitively stimulating activity is associated with better mental functioning.

The use of the internet for study and work shows that it can be a tool for accessing and processing information. We have observed that compared to nonregular use, frequent use the internet for instrumental activities can significantly protect against cognitive decline. Study and work provide cognitive stimuli, and the internet serves as a platform for a cognitive reserve that continually promotes recovery from functional brain damage to prevent or delay the development of cognitive impairment [17,30]. Using the internet to study and work exercises the brain’s ability to collect and process information, increasing the brain’s cognitive reserve, which is associated with better cognitive performance; in addition, the information available on the internet enhances the health literacy of middle-aged and older adults. This study’s results are similar to those obtained in other settings [31]. The WHO has shown the positive impact of good health literacy on mitigating the risk of cognitive decline and dementia in its Guidelines for Mitigating Cognitive Decline and Dementia.

In the study by Kobayashi et al [31], social participation was found to have three components: civic, entertainment, and cultural. It was shown that fair social participation improves the health literacy of middle-aged and older adults. After refining its purpose, we believe that using the internet for socializing and entertainment is a form of social engagement. Socializing and entertainment help people meet their emotional needs. It has been shown that from the perspective of brain evolution and functional development, the need for emotion contributes to the generation of neural networks for specific phenotypes of individual social cognition [32]. As there are many ways to use the internet for entertainment, older people can choose to enrich their lives in interesting and appropriate ways, and an enjoyable experience could bring them satisfaction and a sense of accomplishment. Regarding socializing, previous studies have shown that increased contact with family and friends via the internet by older adults has a positive impact on enhancing both life satisfaction and mood; this is especially true for older adults with health problems and limited ability to perform, since increased contact provides important opportunities to establish and maintain intimate relationships through social networking [33]. In contrast, this study showed that moderate socializing was significantly associated with reduced cognitive decline. Excessive online socializing may be related to an addiction to the emotional connections of the virtual world and a gap in real life, leading to a failure to adapt to stress. This is similar to the results of some studies, for example, Nie et al [34], who concluded that frequency of internet use is significantly negatively related to happiness, and that excessive use of the internet instead of other offline activities produces a negative effect.

Taking part in commercial-related activities was the only internet activity that showed a negative relationship with cognitive function. Internet shopping behavior was chosen for the measurement of commercial-related activities. A study by Holtfreter et al [35] showed that middle-aged and older adults are more likely to be at risk of online fraud, especially those with low self-control who are exposed to a high-risk, online, commercial-related activity environment where both online fraud and identity theft endanger the mental health of middle-aged and older adults. We hypothesized that because commercial-related activities involve money, older adults would be stressed by their infrequent and unskilled online shopping activities. Also, as the main victims of online fraud, older adults would be more cautious and worried about online payments. These emotions are detrimental to the mental health of older adults. The findings showed that when older adults used the internet frequently for commercial-related activities, the adverse effects decreased and were no longer significantly associated with negative cognitive function. We believe that older adults who do not use the internet frequently for commercial-related activities have lower digital literacy. Older adults with lower health literacy are vulnerable to online scams leading to stress adaptation failure. In contrast, older adults who frequently use the internet for commercial-related activities have higher digital literacy and good stress adaptation. Therefore, frequent use of the internet by older adults for commercial-related activities is not associated with a change in cognitive function.

In conclusion, our findings provide some evidence that the use of the internet is essential for middle-aged and older adults, especially for patients with cognitive decline. With the internet as an increasingly popular technological tool whose use is escalating, we can promote its use in the health field for the middle-aged and older adult population. Our findings advance the possibility of internet interventions for cognitive decline. Our findings also allow future research studies to fully consider the differential impact of the type, frequency, and diversity of internet use and to provide practical implementation options for nonpharmacological interventions for cognitive decline.

### Strengths and Limitations

To the best of our knowledge, this study is the first to use a fixed-effects analysis to measure the relationship between internet use and cognitive decline among middle-aged and older adult Chinese people, ruling out potential endogeneity. This study confirms that greater diversity and higher frequency of internet use are associated with alleviating cognitive decline by assessing the net association between changes in internet use and changes in cognitive function. At the same time, this is one of the few studies that has finely delineated the internet use variable to explore the differential relationships of different internet use purposes on cognitive decline. These findings have practical implications and can serve as quantitative criteria to guide the design of intervention programs. The results of this study also remind subsequent researchers to discuss internet use in a disaggregated manner when exploring the causal relationship between internet use and cognitive decline.

The findings should be interpreted with caution because of the following limitations. First, some short-term benefits may diminish over time, given the impact of internet use on cognitive decline over the same period. Second, considering that there were 2 years between each wave of the CFPS study, this study used internet use and cognitive decline in both waves, rather than a lagged model. The sample size in this study is indeed more extensive than in the lagged model. However, the exact causal relationship remains to be further investigated. Third, we can only speculate about the mechanisms applicable to the findings through the observed statistical results, and the observational nature of the study limits our ability to confirm the causal and theoretical mechanisms of internet use and cognitive decline. Fourth, vocabulary is a cognitive skill; however, it is known to be relatively insensitive to cerebral pathology and, thus, unlikely to be a good choice of measures to assess cognitive decline over time. Also, the original questionnaire did not address the duration of internet use. Follow-up research can explore the effects of specific internet usage time. Although our study had a superficial discussion of the topic of mental health, the relationship between cognitive decline, internet use, and mental health was not clarified; in addition, variables measuring the mental health of middle-aged and older adults were not used in the study, and the relationship between the three needs to be further investigated.

### Conclusions

Previous studies using CFPS data have demonstrated that internet use is associated with better mental health in older adults. This study refined the relationship between type, diversity, and frequency of internet use and cognitive decline by expanding internet use dimensions. In summary, there were three main findings from this study. First, using the internet for study, work, entertainment, socializing, and commercial-related activities was associated with varying degrees of alleviated cognitive decline. Second, more frequent use of the internet for study and work was associated with better outcomes, while moderate use of the internet for socializing, entertainment, and commercial-related activities was associated with better outcomes. Finally, there is a relationship between frequency and diversity of internet use and better cognitive function, with both depth and breadth of internet use being important, and with more varied and frequent use of the internet being associated with better outcomes. These findings suggest that different levels of internet use have different relationships with cognitive function in middle-aged and older adults; the findings serve as a reminder of the need for more targeted and quantitatively sound policies when designing nonpharmacological interventions for cognitive decline. Future research directions could target studies of the mechanisms responsible for the differential impact outcomes of internet use.

## Abbreviations

CFPS: China Family Panel Studies
ELSA: English Longitudinal Study of Aging
WHO: World Health Organization
